# Association of IL-10 and IL-10Rβ gene polymorphisms with graft-versus-host disease after haematopoietic stem cell transplantation from an HLA-identical sibling donor

**DOI:** 10.1186/1471-2172-10-24

**Published:** 2009-05-04

**Authors:** Jyrki Sivula, Hannu Turpeinen, Liisa Volin, Jukka Partanen

**Affiliations:** 1 Research and Development, Finnish Red Cross Blood Service, Helsinki, Finland; 2 Department of Medicine, Helsinki University Central Hospital, Helsinki, Finland

## Abstract

**Background:**

Extensive allelic matching in the human leukocyte antigen (HLA) genes is regarded as a prerequisite for good clinical success of allogeneic haematopoietic stem cell transplantation (HSCT). Also other genetic factors can be assumed to play a role in preventing and controlling the complications associated with allogeneic HSCT, in particular graft-versus-host disease (GvHD). Interleukin-10 (IL-10) and its receptor (IL-10R), key regulators of the immune response, are among these candidates. We studied the association of IL-10 and IL-10Rβ gene polymorphisms with the occurrence of GvHD in 309 HLA-identical sibling donor and recipient pairs.

**Results:**

The difference in genotypic IL-10 production between patient and donor in combination with patient IL-10Rβ A/A genotype predisposed strongly to acute GvHD (OR = 7.15, p = 0.000023). On the other hand, a combination of same genotypic IL-10 production with patient IL-10Rβ A/A genotype protected from chronic GvHD (OR = 0.407, p = 0.0097).

**Conclusion:**

Our results suggest that IL-10 and IL-10Rβ genes have a synergistic effect on the risk of GvHD.

## Background

Extensive allelic matching in the human leukocyte antigen (HLA) genes between recipient and donor is regarded as a prerequisite for good clinical success of allogeneic haematopoietic stem cell transplantation (HSCT) [1]. Graft-versus-host disease (GvHD) can not be totally avoided even with fully HLA-matched sibling donors or by using current GvHD prophylaxis, indicating complex and multifactorial nature of GvHD. This has lead into search of novel genetic factors, in addition to the HLA genes, to prevent and predict the occurrence of GvHD. Genes for killer cell immunoglobulin like receptors (KIR), KIR ligands and minor histocompatibility antigens as well as several cytokines and cytokine receptors have obtained a considerable interest [2-4].

Cytokine interleukin 10 (IL-10) is a good functional candidate for GvHD-related gene [5,6]. Previous studies have shown that patient or donor IL-10 genotypes separately associate with both acute and chronic GvHD [6-9]. IL-10 is produced by a variety of different cells, of both haematopoietic and non-haematopoietic lineages [10]. IL-10 is usually regarded as a potent suppressor of the immune responses and hence it is thought to be useful in preventing GvHD. However, it has also been shown to have some immunostimulatory effects [11-13]. Interleukin 10 receptor beta (IL-10Rβ) is also expressed on several cell types, and its role varies depending on the cell type it is expressed on [14]. In haematopoietic cell lineages IL-10Rβ functions as a part of IL-10 receptor together with IL-10 receptor alpha (IL-10Rα). IL-10Rα, the IL-10 specific part of the receptor complex, is only expressed on haematopoietic cells [5]. Thus in haematopoietic cells IL-10Rβ is mediating the IL-10 signal. In many non-haematopoietic cell lineages IL-10Rβ functions as a part of a receptor for other cytokines [14].

To address the role of IL-10 and IL-10Rβ in HSCT we here report the effects of polymorphisms in these genes on the incidence of acute and chronic GvHD in 309 Finnish HSCT recipients who had an HLA identical sibling donor. In particular, we addressed the questions of interplay of patient- and donor-specific factors of IL-10 and IL-10Rβ genes.

## Results

Altogether 309 allogeneic HSCT recipients and their HLA-identical sibling donors were included in the study. The demographic and other background information is given in Table 1. Genetic variation in IL-10 and IL-10Rβ genes was studied and genetic association with the occurrence of GvHD in the patients was tested.

**Table 1:** Sample demographics

***Patient age***, *years mean (range)*	47 (17–66)
***Donor age***, *years mean (range)*	45 (11–68)
***Diagnosis***, *frequency*	
Acute myeloid leukaemia	77
Multiple myeloma	54
Acute lymphoid leukaemia	48
Chronic lymphoid leukaemia	45
Myelodysplastic syndrome	23
Chronic myeloid leukaemia	13
Non-Hodgkin lymphoma	13
Other	36

***Disease status***	
Good prognosis	148
Bad prognosis	142

***Gender***, *patient-donor*	
male-male	82
male-female	67
female-male	76
female-female	63

***Graft origin***	
Peripheral blood	128
Bone marrow	162

***Conditioning***	
Myeloablative	233
Other	57

***GvHD prevention***	
Cyclosporine, Methotrexate and Methylprednisolone	207
Cyclosporine and Mycophenolate Mofetil	46
Cyclosporine and Methotrexate	37

***Acute GvHD***	
No	197
grade III–IV	29

***Chronic GvHD***	
No	117
Extensive	72

### 1. Genotype and haplotype association of IL-10 and IL-10Rβ gene polymorphisms with GvHD

The distributions of genotypes and haplotypes in patients and donors were studied for their association with the occurrence of acute (none versus grade III–IV) and chronic (none versus extensive) GvHD (Additional files 1 and 2).

In the patients, the genotype rs1800872 A/A in IL-10 gene predisposed to grade III–IV acute GvHD (p = 0.031, OR = 3.83) when compared to other genotypes (A/C and C/C). As a result of strong linkage disequilibrium between the IL-10 gene markers, the same association was also detected for the rs1800872 – rs1800896 haplotypes (Additional files 1 and 2). In the IL-10Rβ gene, patient's A/A genotype predisposed to acute GvHD (p = 0.0035, OR = 3.88) and A/G genotype showed protection from it (p = 0.017, OR = 0.30).

When the patients and donors were stratified based on their IL-10 and IL-10Rβ genotypes together, this approach did not suggest any novel genetic association.

In the patients, none of the genotypes of IL-10 or IL-10Rβ showed statistically significant association with chronic GvHD. In the donors, no genotypes or haplotypes showed statistically significant association with either chronic or acute GvHD (Additional files 1 and 2).

Previously, Lin et al. [7] described that a certain combination of patient IL-10 and donor IL-10Rβ polymorphisms protected very strongly from acute GvHD. Hence, we carried out similar analysis (Table 2). We also found that none of the three sibling pairs in our data set with IL-10 rs1800872 A/A (patient) – IL-10Rβ rs28341676 G/G (donor) genotypes actually resulted in acute GvHD, but no statistical significancy or similar trend of decreasing acute GvHD frequency as Lin et al. observed toward this genotype combination could be seen.

**Table 2:** Incidence of grade III–IV acute GvHD according to patient IL-10 (-592) rs1800872 and donor IL-10Rβ(+238) rs28341676 genotypes/all cases (percent)

		**Patient IL-10 rs1800872**		
		
		**A/A**	**A/C**	**C/C**
	
**Donor IL-10Rβ rs28341676**	**A/A**	3/8 (38%)	5/53 (9%)	9/90 (10%)
	**A/G**	2/5 (40%)	4/32 (13%)	4/70 (6%)
	**G/G**	0/3 (0%)	0/12 (0%)	1/24 (4%)

### 2. Associations with predicted IL-10 production levels

Genotype classes with different predicted production levels were tested for their association with the occurrence of GvHD but no statistically significant results could be found. However, patient intermediate genotypic production level was borderline protective from acute GvHD (p = 0.073, OR = 0.431).

We furthermore subdivided the study group based on their IL-10Rβ genotype and whether the donor – recipient pair had the same or different production level of IL-10 (Additional file 3). Those patients with the IL-10Rβ A/A genotype who received the graft from a donor with a different predicted IL-10 level to that of the recipient, had a very high risk for acute GvHD (p = 0.000023, OR = 7.15). Similarly, when the IL-10Rβ A/A homozygous patients received a graft with a same IL-10 production level, they were protected from chronic GvHD (p = 0.0097, OR = 0.407; Additional file 3).

### 3. Multivariate analysis

Logistic regression multivariate analysis was then performed to test if the statistically significant results observed in univariate analyses would hold, when tested in the context of other known factors influencing GvHD; disease status (good prognosis versus bad prognosis), gender match (male versus female donor), graft origin (bone marrow versus peripheral blood), pre-transplantation conditioning (myeloablative versus other) and aGvHD as a risk factor for cGvHD. All statistically significant results of univariate IL-10 and IL-10Rβ remained significant in multivariable analyses. Results of these analyses are shown in Table 3. We also performed multivariable analyses to see if the studied IL-10 and IL-10Rβ polymorphisms correlated with the other factors used in these analysis. These results showed no correlation between disease status, gender match, graft origin and pre-transplantation conditioning, and the IL-10 and IL-10Rβ polymorphisms.

**Table 3:** Multivariable logistic regression analysis of acute and chronic GvHD

Acute GvHD	B	S.E.	p
Disease status	0.92	0.47	0.053
Gender match	-0.29	0.50	0.56
Graft origin	0.34	0.50	0.49
Conditioning	0.055	0.60	0.93
Patient IL10 rs1800872 A/A	1.30	0.60	0.031
Constant	-2.65	0.53	

Acute GvHD	B	S.E.	p

Disease status	1.26	0.53	0.017
Gender match	-0.32	0.50	0.53
Graft origin	0.13	0.49	0.80
Conditioning	-0.11	0.61	0.85
Patient IL10Rβ rs28341676 A/A	1.33	0.46	0.0041
Constant	-3.13	0.58	

Acute GvHD	B	S.E.	p

Disease status	1.26	0.53	0.017
Gender match	-0.39	0.56	0.49
Graft origin	0.09	0.52	0.86
Conditioning	-0.52	0.67	0.44
IL-10 production level different and patient IL-10Rβ A/A	2.16	0.49	0.0000088
Constant	-3.02	0.56	

Chronic GvHD	B	S.E.	p

Disease status	0.38	0.50	0.50
Gender match	-1.20	0.55	0.028
Graft origin	-1.58	0.48	0.0010
Conditioning	1.10	0.65	0.087
aGvHD	2.64	0.95	0.0052
IL-10 production level same and patient IL-10Rβ A/A	-1.18	0.50	0.017
Constant	0.58	0.43	

## Discussion

In the present study we analysed the role of IL-10 and IL-10Rβ SNPs on the incidence of acute and chronic GvHD after HSCT between HLA-identical siblings. Previously published studies on interaction of IL-10 and HSCT have found association between IL-10 polymorphism and GvHD outcome [6,8,9,15-18]. In these studies the results predominantly rely on polymorphisms of either recipient or donor. Here we addressed the question also by studying the association of the SNPs with GvHD in biologically more meaningful context; that is analysing simultaneously both donor and patient genotypes.

In our analysis patient IL-10 rs1800872 genotype A/A was associated with worse acute GvHD outcome (Additional file 1 and Table 3). Similar result was also observed with rs1800896 and rs1800872 genotype combination AA/AA. Also patient IL-10Rβ rs28341676 genotype affected the risk of developing GvHD. Patient IL-10Rβ rs28341676 A/A predisposed to and A/G protected from grades III–IV acute GvHD. Donor IL-10 rs1800896 and rs1800872 or IL-10Rβ rs28341676 polymorphisms did not have any clear effect on the GvHD outcome (Additional file 2).

Because the cells of the graft are mainly haematopoietic cells, the effect of IL-10Rβ polymorphism on the donor side is restricted to recognition of IL-10. Since most of the patient's original haematopoietic cells are eliminated, the effect of IL-10Rβ on patient side is largely restricted to the signalling of other cytokines where the IL-10Rβ also functions as a part of the receptor complex. Cytokines known to date to utilise IL-10Rβ are IL-10, IL-22, IL-26, IL-28A, IL-28B and IL-29 [14]. Since donor IL-10Rβ had no significance on GvHD predisposition, the effects of IL-10Rβ polymorphism would seem to rely largely on functions of these other cytokines utilizing IL-10Rβ in their signalling.

When analysing both the patient and donor IL-10 genotypes the results showed a trend for lower risk of developing GvHD when both had similar IL-10 production levels estimated from genotypes (Additional file 3). Similar results were also seen by Bertinetto et al [18]. In their study the presence of IL-10 rs180087 G allele in both patient and donor was associated with a trend for lower risk of developing GvHD. The rationale for this is the fact that IL-10 is a strong regulator of immune system and it might be that either higher or lower production level of the protein as compared to original one would not be optimal for the patient's immune system. The effect of patient IL-10Rβ was also amplified when analyzed together with IL-10 production level. IL-10Rβ A/A strongly predisposed to acute GvHD when patient and donor IL-10 production level differed, but protected from chronic GvHD when patient and donor had similar IL-10 production level (Additional file 3 and Table 3).

We were not able to reproduce most of the significant results of Lin et al [7] with our study material. In our results the donor IL-10Rβ had no significant effect on the GvHD even in combination with IL-10 polymorphisms. Also, patient IL-10 rs1800872 A/A predisposed to acute GvHD (Additional file 1 p = 0.031, OR = 3.83). However, as Lin et al also observed, no acute GvHD cases were present among those three pairs with the combination of patient IL-10 rs1800872 A/A and donor IL-10Rβ rs28341676 G/G (Table 2). Compared to our results, in Lin et al study the frequency of IL-10 rs1800872 A allele was higher. This might in part explain the striking difference between our results. The rs1800872 A allele is often associated with lower risk of developing GvHD, but there are also studies which show similar association as we found [6,18]. It is possible that the rs1800872 is not it self affecting to the risk of GvHD, but is in linkage disequilibrium with the actual effector. In different populations this linkage might vary and cause the discrepancy in the results.

In analyses of IL-10Rβ rs28341676 the results differed between acute and chronic GvHD. Patient IL-10Rβ A/A predisposed to acute GvHD and seemed to protect from chronic GvHD. The different effect of IL-10Rβ polymorphism between acute GvHD and chronic GvHD and overall weaker results in chronic GvHD can be understood by the immunologic differences between acute and chronic GvHD. During the period of which acute GvHD is observed, the immune system is just starting to reconstitute anew after the HSCT. Also, many of the effector cells had been developed within the donor. In many cell lineages their numbers start rising only well after the usual 100 days time limit of acute GvHD diagnosis. More cells of donor origin are developing within the recipient during chronic GvHD. The immune system is more mature and complex than in the newly transplanted patient [19]. Notably, amounts of regulatory cells rise only relatively late in immune reconstitution [20]. The full reconstitution of immune system after transplantation may take years. Hence it is not surprising that IL-10 and IL-10Rβ polymorphisms have less effect in chronic GvHD than in acute GvHD. This could also explain the opposing effect of IL-10Rβ polymorphism in acute GvHD and chronic GvHD.

We are fully aware of the possible problems in analyzing all the transplantations as a single group. Grafting peripheral blood stem cells instead of bone marrow cells predisposes to higher incidence of chronic GvHD. Also pre-treatment conditioning, fully myeloablative versus reduced intensity conditioning, highly affects the complications after HSCT. To address this issue we performed logistic regression multivariate analysis to test the results of univariate analyses in the context of known GvHD affecting factors; disease status, gender match, graft origin, pre-transplantation conditioning and aGvHD as a risk factor for cGvHD. All the statistically significant results from univariate analyses remained significant in the multivariate analyses (Table 3). The p-values in this study were not corrected for multiple comparisons. These results need to be validated in other cohorts and further studies are also needed to clarify the relationships between IL-10 and IL-10Rβ.

## Conclusion

We didobserve synergistic effect between IL-10 and IL-10Rβ in predisposition to GvHD. The effect of IL-10Rβ polymorphism was strengthened when it was analysed together with IL-10 polymorphisms. In our samples similarity of IL-10 production level was associated with lower incidence of both acute and chronic GvHD. Different effect of IL-10Rβ genotype in acute GvHD and chronic GvHD could be explained by the developing immune system, arise of new regulatory cells and passing cytokine storm associated with the transplantation.

## Methods

### Patients

Altogether 309 adult allogeneic HSCT recipients and their HLA-identical sibling donors were included in this retrospective study. All transplantations were performed in a single centre (Department of Medicine, Helsinki University Central Hospital, Helsinki, Finland) between years 1993 and 2005. Clinical data were collected on diagnosis, age, gender match, transplantation protocol, date of transplantation and acute GvHD and chronic GvHD manifestation. GvHD was diagnosed and graded according to standard criteria [21]. Patient and donor descriptive demographics are shown in Table 1.

This study was approved by the Ethical Review Board of the Helsinki University Hospital, Helsinki, Finland.

### IL-10 and IL-10Rβ genotyping

Two single nucleotide polymorphisms (SNPs) in IL-10 and one in IL-10Rβ were genotyped. The IL-10 SNPs at locations -1082 (rs1800896) and -592 (rs1800872) and the IL-10Rβ SNP at location +238 (rs28341676) were typed using restriction fragment length polymorphism method as described earlier [7,22]. EcoNI recognition site primers and restriction enzyme was used for IL-10.

In the Finnish population, the selected two markers for IL-10 tag completely the often used three-marker haplotype (-1082 A/G (rs1800896), -819 T/C (rs1800871), and -592 A/C (rs1800872)) for the gene [23].

### Statistical analysis

To analyse the effect of IL-10 and IL-10Rβ polymorphisms on the GvHD outcome we used different combinations of patient and donor genotyping data. We (i) analysed IL-10 and IL-10Rβ genotypes and haplotypes separately in patients and donors, and (ii) combined the genotyping data from each patient-donor pair. We also (iii) grouped the IL-10 genotypes into three production level groups: high, intermediate and low producing. These groups are based on the effect of IL-10 polymorphisms on the level of protein production as published earlier [24,25]: IL-10 rs1800896 (SNP1) and rs1800872 (SNP2) genotype combination (SNP1SNP2/SNP1SNP2) GC/GC belongs to high producing, GC/AC and GC/AA to intermediate producing, and AC/AC, AC/AA and AA/AA to low producing group.

Analyses of different IL-10 and IL-10Rβ combinations were done in relation to the acute GvHD and chronic GvHD incidence using χ^2 ^or Fisher's exact tests. Recipients with acute GvHD of grade III–IV and their donors formed the positive group which was compared to patients with no acute GvHD and their donors. Chronic GvHD positive group patients had extensive chronic GvHD and their controls had no chronic GvHD. To test the statistically significant results of univariate analyses in the context of other risk factors we constructed a multivariable logistic regression model adjusted for disease status, gender match, graft origin and pre-transplantation conditioning. We also included aGvHD as a risk factor for cGvHD. The SPSS for Windows v15.0 software (SPSS Inc., Chicago, IL, USA) was used for the analyses. All p-values are considered as two-sided and uncorrected for multiple testing. P-values under 0.05 were regarded to indicate statistical significance.

## Abbreviations

GvHD: Graft versus host disease; aGvHD: Acute graft versus host disease; cGvHD: Chronic graft versus host disease; HLA: Human leukocyte antigen; HSCT: Haematopoietic stem cell transplantation; IL-10: Interleukin-10; IL-10R: Interleukin-10 receptor; IL-10Rβ: Interleukin-10 receptor beta subunit; IL-10Rα: Interleukin-10 receptor alpha subunit; KIR: Killer cell immunoglobulin like receptor.

## Competing interests

The authors declare that they have no competing interests.

## Authors' contributions

LV clinical expertise, JS and HT genetic typing and data analysis. All authors contributed in the study hypothesis and study design and all authors contributed to the preparation of the manuscript. All authors read and approved the final manuscript. JS and HT share an equal contribution to the article.

## Authors' information

JS, HT and JP: Research and Development, Finnish Red Cross Blood Service, Helsinki, Finland

LV: Department of Medicine, Helsinki University Central Hospital, Helsinki, Finland

Address for correspondence: Dr Jukka Partanen, Research and Development, Finnish Red Cross Blood Service, Kivihaantie 7, 00310 Helsinki, Finland. jukka.partanen@bts.redcross.fi

## Supplementary Material

Additional file 1 **Table s1**. Distribution of IL-10 (-1082) rs1800896 and (-592) rs1800872, and IL-10Rβ (+238) rs28341676 genotypes and their association with the occurrence of acute and chronic GvHD in the patients Click here for file

Additional file 2 **Table s2**. Distribution of IL-10 (-1082) rs1800896 and (-592) rs1800872, and IL-10Rβ (+238) rs28341676 genotypes and their association with the occurrence of acute and chronic GvHD in the patients donors Click here for file

Additional file 3 **Table s3**. Incidence of GvHD according to patient IL-10Rβ (+238) rs28341676 and patient and donor IL-10 genotypic production levels Click here for file
