# Supplementary material for: Association of IL-10 and IL-10Rβ gene polymorphisms with graft-versus-host disease after haematopoietic stem cell transplantation from an HLA-identical sibling donor
Source: BMC Immunol. 2009 May 4;10:24. doi: 10.1186/1471-2172-10-24 (PMC2685414; doi:10.1186/1471-2172-10-24)
Supplement: Additional file 1 — Table s1. Distribution of IL-10 (-1082) rs1800896 and (-592) rs1800872, and IL-10Rβ (+238) rs28341676 genotypes and their association with the occurrence of acute and chronic GvHD in the patients [file 1471-2172-10-24-S1.doc]

Table 2. Distribution of IL-10 (-1082) rs1800896 and (-592) rs1800872, and IL-10Rβ (+238) rs28341676 genotypes and their association with the occurrence of acute and chronic GvHD in the patients.

| Genotype | N | % | Acute GvHD | | | | Chronic GvHD | | |  |
| --- | --- | --- | --- | --- | --- | --- | --- | --- | --- | --- |
|  |  |  | None | III-IV | OR | p | None | Extensive | OR | p |
| IL-10 rs1800896 A/A | 86 | 28.3 | 55(64%) | 10(12%) |  | n.s. | 29(34%) | 21(24%) |  | n.s. |
| IL-10 rs1800896 A/G | 155 | 51.0 | 101(65%) | 9(6%) |  | n.s. | 63(41%) | 37(24%) |  | n.s. |
| IL-10 rs1800896 G/G | 63 | 20.7 | 38(60%) | 10(16%) |  | n.s. | 24(38%) | 13(21%) |  | n.s. |
|  |  |  |  |  |  |  |  |  |  |  |
| IL-10 rs1800872 A/A | 18 | 5.9 | 10(56%) | 5(28%) | 3.83 | 0.031 | 4(22%) | 3(17%) |  | n.s. |
| IL-10 rs1800872 A/C | 100 | 32.9 | 64(64%) | 10(10%) |  | n.s. | 43(43%) | 25(25%) |  | n.s. |
| IL-10 rs1800872 C/C | 186 | 61.2 | 120(65%) | 14(8%) |  | n.s. | 70(38%) | 42(23%) |  | n.s. |
|  |  |  |  |  |  |  |  |  |  |  |
| IL-10 AA/AA | 17 | 5.7 | 9(53%) | 5(29%) | 4.40 | 0.021 | 4(24%) | 3(18%) |  | n.s. |
| IL-10 AA/AC | 38 | 12.7 | 26(68%) | 4(11%) |  | n.s. | 16(42%) | 10(26%) |  | n.s. |
| IL-10 AC/AC | 31 | 10.3 | 20(65%) | 1(3%) |  | n.s. | 9(29%) | 8(26%) |  | n.s. |
| IL-10 AA/GC | 60 | 20.0 | 37(62%) | 5(8%) |  | n.s. | 26(43%) | 14(23%) |  | n.s. |
| IL-10 AC/GC | 94 | 31.3 | 63(67%) | 4(4%) |  | n.s. | 37(39%) | 23(24%) |  | n.s. |
| IL-10 GC/GC | 60 | 20.0 | 36(60%) | 9(15%) |  | n.s. | 23(38%) | 11(18%) |  | n.s. |
|  |  |  |  |  |  |  |  |  |  |  |
| Low genotypic IL-10 production | 86 | 28.7 | 55(64%) | 10(12%) |  | n.s. | 29(34%) | 21(24%) |  | n.s. |
| Intermediate genotypic IL-10 production | 154 | 51.3 | 100(65%) | 9(6%) |  | n.s. | 63(41%) | 37(24%) |  | n.s. |
| High genotypic IL-10 production | 60 | 20.0 | 36(60%) | 9(15%) |  | n.s. | 23(38%) | 11(18%) |  | n.s. |
|  |  |  |  |  |  |  |  |  |  |  |
| IL-10Rβ rs28341676 A/A | 138 | 46.3 | 85(62%) | 22(16%) | 3.88 | 0.0035 | 56(41%) | 26(19%) |  | n.s. |
| IL-10Rβ rs28341676 A/G | 133 | 44.6 | 88(66%) | 6(5%) | 0.302 | 0.017 | 47(35%) | 38(29%) |  | n.s. |
| IL-10Rβ rs28341676 G/G | 27 | 9.1 | 17(63%) | 1(4%) |  | n.s. | 10(37%) | 5(19%) |  | n.s. |

IL-10 two SNP haplotype in the third section of this table is designated as (rs1800896,rs1800872)/(rs1800896,rs1800872). Percentages in the parenthesis are from all the cases in the same genetic category.
