# Supplementary material for: Association of IL-10 and IL-10Rβ gene polymorphisms with graft-versus-host disease after haematopoietic stem cell transplantation from an HLA-identical sibling donor
Source: BMC Immunol. 2009 May 4;10:24. doi: 10.1186/1471-2172-10-24 (PMC2685414; doi:10.1186/1471-2172-10-24)
Supplement: Additional file 2 — Table s2. Distribution of IL-10 (-1082) rs1800896 and (-592) rs1800872, and IL-10Rβ (+238) rs28341676 genotypes and their association with the occurrence of acute and chronic GvHD in the patients donors [file 1471-2172-10-24-S2.doc]

Table 3. Distribution of IL-10 (-1082) rs1800896 and (-592) rs1800872, and IL-10Rβ (+238) rs28341676 genotypes and their association with the occurrence of acute and chronic GvHD in the patients donors.

| Genotype | N | % | Acute GvHD | | | | Chronic GvHD | | |  |
| --- | --- | --- | --- | --- | --- | --- | --- | --- | --- | --- |
|  |  |  | None | III-IV | OR | p | None | Extensive | OR | p |
| IL-10 rs1800896 A/A | 85 | 27.5 | 55(65%) | 10(12%) |  | n.s. | 29(34%) | 21(25%) |  | n.s. |
| IL-10 rs1800896 A/G | 158 | 51.1 | 101(64%) | 9(6%) |  | n.s. | 63(40%) | 37(23%) |  | n.s. |
| IL-10 rs1800896 G/G | 66 | 21.4 | 38(58%) | 10(15%) |  | n.s. | 24(36%) | 13(20%) |  | n.s. |
|  |  |  |  |  |  |  |  |  |  |  |
| IL-10 rs1800872 A/A | 17 | 5.5 | 10(59%) | 5(29%) |  | n.s. | 4(24%) | 3(18%) |  | n.s. |
| IL-10 rs1800872 A/C | 103 | 33.3 | 64(62%) | 10(10%) |  | n.s. | 43(42%) | 25(24%) |  | n.s. |
| IL-10 rs1800872 C/C | 189 | 61.2 | 120(63%) | 14(7%) |  | n.s. | 70(37%) | 42(22%) |  | n.s. |
|  |  |  |  |  |  |  |  |  |  |  |
| IL-10 AA/AA | 17 | 5.5 | 12(71%) | 3(18%) |  | n.s. | 7(41%) | 2(12%) |  | n.s. |
| IL-10 AA/AC | 34 | 11.0 | 20(59%) | 3(9%) |  | n.s. | 15(44%) | 12(35%) |  | n.s. |
| IL-10 AC/AC | 34 | 11.0 | 23(68%) | 1(3%) |  | n.s. | 12(35%) | 9(26%) |  | n.s. |
| IL-10 AA/GC | 69 | 22.3 | 41(59%) | 9(13%) |  | n.s. | 25(36%) | 12(17%) |  | n.s. |
| IL-10 AC/GC | 89 | 28.8 | 55(62%) | 4(4%) |  | n.s. | 29(33%) | 23(26%) |  | n.s. |
| IL-10 GC/GC | 66 | 21.4 | 46(70%) | 9(14%) |  | n.s. | 29(44%) | 14(21%) |  | n.s. |
|  |  |  |  |  |  |  |  |  |  |  |
| Low genotypic IL-10 production | 85 | 27.5 | 55(65%) | 7(8%) |  | n.s. | 34(40%) | 23(27%) |  | n.s. |
| Intermediate genotypic IL-10 production | 158 | 51.1 | 96(61%) | 13(8%) |  | n.s. | 54(34%) | 35(22%) |  | n.s. |
| High genotypic IL-10 production | 66 | 21.4 | 46(70%) | 9(14%) |  | n.s. | 29(44%) | 14(21%) |  | n.s. |
|  |  |  |  |  |  |  |  |  |  |  |
| IL-10Rβ rs28341676 A/A | 154 | 51.2 | 101(66%) | 17(11%) |  | n.s. | 58(38%) | 33(21%) |  | n.s. |
| IL-10Rβ rs28341676 A/G | 108 | 35.9 | 69(64%) | 10(9%) |  | n.s. | 42(39%) | 25(23%) |  | n.s. |
| IL-10Rβ rs28341676 G/G | 39 | 13.0 | 24(62%) | 1(3%) |  | n.s. | 16(41%) | 9(23%) |  | n.s. |

IL-10 two SNP haplotype in the third section of this table is designated as (rs1800896,rs1800872)/(rs1800896,rs1800872). Percentages in the parenthesis are from all the cases in the same genetic category.
