# Supplementary material for: Association of IL-10 and IL-10Rβ gene polymorphisms with graft-versus-host disease after haematopoietic stem cell transplantation from an HLA-identical sibling donor
Source: BMC Immunol. 2009 May 4;10:24. doi: 10.1186/1471-2172-10-24 (PMC2685414; doi:10.1186/1471-2172-10-24)
Supplement: Additional file 3 — Table s3. Incidence of GvHD according to patient IL-10Rβ (+238) rs28341676 and patient and donor IL-10 genotypic production levels [file 1471-2172-10-24-S3.doc]

Table 5. Incidence of GvHD according to patient IL-10Rβ (+238) rs28341676 and patient and donor IL-10 genotypic production levels.

|  | IL-10 | N | % | Acute GvHD | | | | Chronic GvHD | | | |
| --- | --- | --- | --- | --- | --- | --- | --- | --- | --- | --- | --- |
|  | production level |  |  | No | III-IV | OR | p | No | Extensive | OR | p |
|  | Same | 190 | 63.3 | 120(63%) | 13(7%) |  | n.s. | 79(42%) | 39(21%) |  | n.s. |
|  | Different | 110 | 37.7 | 71(65%) | 15(14%) |  | n.s. | 36(33%) | 30(27%) |  | n.s. |
|  |  |  |  |  |  |  |  |  |  |  |  |
| Patient | IL-10 |  |  | Acute CvHD | | | | Chronic GvHD | | | |
| IL-10Rβ | production level |  |  | No | III-IV | OR | p | No | Extensive | OR | p |
| A/A | Same | 96 | 33.0 | 64(67%) | 9(9%) |  | n.s. | 46(48%) | 15(16%) | 0.407 | 0.0097 |
|  | Different | 41 | 14.1 | 20(49%) | 13(32%) | 7.15 | 0.000023 | 10(24%) | 11(27%) |  | n.s. |
| A/G | Same | 72 | 24.7 | 45(63%) | 3(4%) |  | n.s. | 25(35%) | 20(28%) |  | n.s. |
|  | Different | 56 | 19.2 | 39(70%) | 2(4%) |  | n.s. | 20(36%) | 16(29%) |  | n.s. |
| G/G | Same | 16 | 5.5 | 8(50%) | 1(6%) |  | n.s. | 6(38%) | 3(19%) |  | n.s. |
|  | Different | 10 | 3.4 | 9(90%) | 0 |  | n.s. | 4(40%) | 2(20%) |  | n.s. |

Percentages in the parenthesis are from all the cases in the same genetic category.
